# Unveiling metabolic pathways involved in the extreme desiccation tolerance of an Atacama cyanobacterium

**DOI:** 10.1038/s41598-023-41879-8

**Published:** 2023-09-22

**Authors:** Rachel A. Moore, Armando Azua-Bustos, Carlos González-Silva, Christopher E. Carr

**Affiliations:** 1 School of Earth and Atmospheric Sciences, Georgia Institute of Technology, 275 Ferst Dr. NW, Atlanta, GA 30332 USA; 2 Centro de Astrobiología (CSIC-INTA), Madrid, Spain; 3 Instituto de Ciencias Biomédicas, Facultad de Ciencias de la Salud, Universidad Autónoma de Chile, Santiago, Chile; 4 Facultad de Ciencias, Universidad de Tarapacá, Arica, Chile; 5 Daniel Guggenheim School of Aerospace Engineering, Georgia Institute of Technology, Atlanta, GA 30332 USA

**Keywords:** Microbial ecology, Computational models, Microbiology, Environmental microbiology

## Abstract

*Gloeocapsopsis dulcis* strain AAB1 is an extremely xerotolerant cyanobacterium isolated from the Atacama Desert (i.e., the driest and oldest desert on Earth) that holds astrobiological significance due to its ability to biosynthesize compatible solutes at ultra-low water activities. We sequenced and assembled the *G. dulcis* genome de novo using a combination of long- and short-read sequencing, which resulted in high-quality consensus sequences of the chromosome and two plasmids. We leveraged the *G. dulcis* genome to generate a genome-scale metabolic model (*iGd895*) to simulate growth in silico*. iGd895* represents, to our knowledge, the first genome-scale metabolic reconstruction developed for an extremely xerotolerant cyanobacterium. The model's predictive capability was assessed by comparing the in silico growth rate with in vitro growth rates of *G. dulcis*, in addition to the synthesis of trehalose. *iGd895* allowed us to explore simulations of key metabolic processes such as essential pathways for water-stress tolerance, and significant alterations to reaction flux distribution and metabolic network reorganization resulting from water limitation. Our study provides insights into the potential metabolic strategies employed by *G. dulcis*, emphasizing the crucial roles of compatible solutes, metabolic water, energy conservation, and the precise regulation of reaction rates in their adaptation to water stress.

## Introduction

*Gloeocapsopsis dulcis* strain AAB1 is a hypolithic and extremely xerotolerant cyanobacterium first identified in biofilms collected from the underside of translucent quartz stones in the Atacama Desert’s Coastal Range^1^. Azua-Bustos et al.^1^ suggested that the hypolithic biofilms rely on the regular coastal fogs, known as camanchacas, as their main source of water. Specifically, *G. dulcis* was shown^2^ to tolerate desiccating conditions in-part by increasingly synthesizing the compatible solutes trehalose and sucrose following several weeks to months of desiccation at the extremely low sustained water activity (a_w_) of 0.4.

The accumulation of compatible solutes is one of the many adaptation mechanisms involved in microbial xerotolerance^3^. Bacteria can biosynthesize or scavenge these solutes from the environment to maintain osmotic equilibrium and protein function^4,5^. Other cellular adjustments due to xeric stress may include changes to the fluidity of the cell membrane, upregulation of DNA-repair proteins and reactive oxygen species scavengers, potential dormancy or sporulation, and an up- or down-regulation of certain metabolic pathways depending on changes in energy demands^6^. The specific metabolic adaptations employed by extremely desiccation-tolerant cyanobacteria like *G. dulcis* remain relatively unknown. However, understanding these adaptations could be critical in identifying strategies for the survival of life in arid planetary environments such as Mars, especially given that the Atacama Desert is an established Martian analog^7^.

In this study, we developed a genome-scale model (GEM; *iGd895*) to explore the metabolic capacity of *G. dulcis* undergoing desiccation. GEMs have been used to decipher microbial metabolism by integrating genomic information into a mathematical framework that simulates metabolism using optimization techniques, such as flux balance analysis (FBA)^8^. This technique allows for the prediction of cellular growth and production rates of metabolites under specified conditions. GEMs have been utilized for diverse applications, such as predicting enzyme functions, modeling microbial cell interactions^9^, and investigating microbial growth in various environmental contexts^10,11^.

We simulated *iGd895* growth using FBA techniques and the COMparision of METabolic states pipeline (ComMet) to elucidate the potential metabolic strategies employed by *G. dulcis* undergoing desiccation. Through the utilization of these techniques, we identified crucial metabolic pathways and reactions potentially involved in desiccation tolerance. Additionally, we determined how these pathways may undergo shifts in response to water limitation. By shedding light on the metabolic adaptations employed by an extremely xerotolerant cyanobacterium, our findings provide not only new insights into the potential mechanisms of desiccation tolerance but also hold promise for the search for microbial life in similar arid environments.

## Methods

### DNA extraction and sequencing

DNA was extracted directly from dried *G. dulcis* cells that were originally isolated by Azua-Bustos et al.^2^ from the Atacama Coastal Range. Briefly, 32 mg of the dried cells were hydrated with 300 µl of phosphate-buffered saline, vortexed for two minutes, and extracted using the Quick-DNA Fecal/Soil Microprep Kit (D6012, Zymo Research, Irvine, CA). The extraction was performed according to manufacturer’s instructions, except for the lysis step, which was performed using a TerraLyzer Cell Disrupter (Zymo Research) for 3 min. DNA was eluted in 20 µl and determined by a fluorometric assay (ThermoFisher Scientific, Qubit™ dsDNA HS Assay Kit Q32854) to have a concentration of 6.2 ng µl^−1^.

An Illumina paired-end sequencing library was created with the Nextera XT DNA library preparation kit (FC-131-1024, Illumina, San Diego, CA), following the manufacturer’s instructions. The library was sequenced on a HiSeq instrument (Illumina, San Diego, CA) in a 2 × 250 paired-end run (PE250). Following sequencing, 6,867,392 Illumina raw read pairs were obtained.

To prepare for ONT sequencing, DNA was extracted using the Quick-DNA Fungal/Bacterial Kit (D6007, Zymo Research, Irvine, CA) by adding five mg of dried cells and 200 µl of sterile deionized water directly to BashingBead tubes. Cell lysis was performed for 2 min using the Terralyzer Cell Disruptor, with a total of 23 mg lysed over five replicates. Replicates were pooled, and 500 ng of the extracted DNA was used to generate ONT sequencing libraries with the 1D ligation method (SQK-LSK108) and native barcoding (EXP-NBD103, Oxford Nanopore Technologies, Oxford, UK) using a “one-pot” barcoding protocol^12^.

ONT sequencing was performed with a SpotON flow cell (R9.4.1) and MinKNOW software 1.14.1 (GUI 2.1.14) (Oxford Nanopore Technologies). Following sequencing, 8302 long-reads were obtained, of which 8105 passed the pre-set filter (i.e., quality score > 7). Raw reads were rebasecalled using the “Super-Accurate” (SUP) model Guppy basecaller (v6.0.1) integrated in MinKNOW prior to sequence assembly.

### Sequence assembly and analysis

The genome sequence was assembled de novo using the Trycycler v0.5.3 hybrid assembly pipeline^13^. First, the ONT reads were subjected to quality control using Filtlong v0.2.1^14^. The reads were subsampled with Trycycler to create 12 read sets. Twelve assemblies were generated from the read sets using the Flye v2.9^15^ and Raven v1.8.1^16^ assemblers (i.e., six assemblies were made with each assembler). The assemblies were visually inspected using Bandage^17^.

The contigs were clustered and reconciled using Trycycler’s cluster and reconcile commands, respectively. This resulted in three clusters: one representing the main chromosome, and the other two representing each plasmid. ONT reads were then partitioned between the reconciled clusters using Trycycler’s partition command. Finally, Trycycler’s consensus command was used to make the consensus sequences for each of the three clusters.

Next, Illumina reads were trimmed with Trimmomatic^18^, which dropped 0.38% of the total reads (i.e., 26,287). The surviving 6,260,175 Illumina read pairs were quality controlled using fastp^19^. The Illumina reads were then used to polish the ONT consensus sequences in two rounds; first with Polypolish v0.5.0-1^20^, and then again with POLCA^21^. Sequence quality, completeness, and potential contamination were checked using the CheckM lineage workflow^22^. The online RAST (Rapid Annotation using Subsystem Technology) service was used to annotate the genome^23^. We also used Prokka to verify gene locations within the main chromosome^24^. The annotated genome was visualized using the online Proksee tool (https://proksee.ca/). The consensus sequences (i.e., chromosome and plasmids) were deposited in the National Center for Biotechnology Information (NCBI) under BioProject PRJNA941297.

### In vitro culturing, optical density, and cell counts of G. dulcis

*Gloeocapsopsis dulcis* cells, provided by Dr. Armando Azua-Bustos^2^, were grown in 250 ml flasks with 100 ml of sterile BG-11 medium (UTEX). Growth assays were conducted in triplicate with the addition of a negative control flask filled with 100 ml of sterile BG-11 medium. The flasks were incubated at 28 °C over several weeks with a 12 h on/off light cycle. Two mL of sample were removed from each flask and placed into semi-micro polystyrene cuvettes (BrandTech, Cat. No. 759075D) to determine the optical density (OD) at 600, 730, and 750 nm over several weeks using a NanoDrop One^C^ instrument (ThermoFisher Scientific, Cat. No. ND-ONE-W).

Following OD measurements, *G. dulcis* cells were counted using a light microscope and C-Chip Neubauer Improved hemocytometers (Digital Bio, Cat. No. DHC-N03). Samples at an OD_730–750_ of 0.1 were homogenized via vortex, and 10 µL were pipetted into each side of the hemocytometer. Cells within the five large squares (area of 1.0 mm^2^) of the hemocytometer were counted, and the average was used to determine cell concentration. The data was plotted in R, and the package Growthcurver^25^ was used to fit a linear regression model and determine the specific growth rate (µ).

### Reconstruction and manual curation of the metabolic network

The draft reconstruction was created in KBase using the “Build Metabolic Model” app and the RAST-annotated genome^26^. KBase built the draft biomass objective function based on its Gram-negative biomass template, which assumes a DNA, RNA, protein, cell wall, lipid, and cofactor fraction of the biomass as 0.026, 0.0655, 0.5284, 0.25, 0.075, and 0.1, respectively. The draft model was gapfilled in KBase on “complete media”, and then downloaded in SBML and Excel formats for manual curation. In Python, the biomass objective function was updated based on the genome and cyanobacterial biomass compositions (i.e., from Nogales et al.^27^ and Shastri and Morgan^28^) using the COBRApy library^29^ to reflect a biomass fractions of DNA, RNA, protein, cell wall, lipid, cofactors, carbohydrates, and other as 0.031, 0.17, 0.51, 0.06, 0.12, 0.04, 0.034, and 0.003, respectively. Growth-associated maintenance energy, deoxyribonucleotides, ribonucleotides, amino acids, cofactors, and inorganic ions (mmol gDW^−1^) were determined using the methods of Thiele and Palsson^30^. The specific biomass composition calculations are detailed in File S1. The flux of pigments in the biomass objective were included based on Toyoshima et al.^31^.

Following that, 45 photosynthesis-related reactions were added to the model (File S1). The reactions were originally developed by Yoshikawa et al.^33^ and later refined by Toyoshima et al.^31^ to model the photosynthetic metabolism of cyanobacteria under different spectral lights (e.g., Akimoto et al.^32^). Incident photon sites were described as reactions R0001, R0010, and R0022, for the phycobilisome, photosystem II, and photosystem I, respectively. State transitions, or the energy transfer from the phycobilisome to photosystems I and II, were described as reactions R0006 and R0007, respectively. We used the incident photon ratio of *Synechocystis* grown under white light as the flux of the photon incident sites^31^. The photon incident rate for the simulation was normalized to 495 mmol gDW^−1^ h^−1^ as per the rationale explained below.

The total cellular surface area per gram of *G. dulcis* biomass was estimated as follows: a spherical *G. dulcis* cell is 3.3 µm diameter^34^, with a volume of 19 µm^−3^, and surface area of 34 µm^2^. We estimated a dry cell weight of 7.4 pg by assuming that 70% of the cell was water^35,36^, and that the remaining ~ 1/3 of cellular components had a density of 1.3 g cm^−3^^37^. Thus, the surface area per gram dry weight of biomass was estimated as 4.59 m^2^. From there, a photon uptake rate of 495 mmol gDW^−1^ h^−1^ could be converted to 30 μE m^−2^ s^−1^, which is the average photosynthetic photon flux density experienced by *G. dulcis* living under quartz rocks, and in the laboratory^1^.

Reactions were manually curated according to Thiele and Palsson^30^. Briefly, confidence scores were assigned to each reaction based on what level of evidence is there to support inclusion of the biochemical reaction. Biochemical, genetic, or sequence/physiological data would receive a confidence score of 4, 3, or 2, respectively. Much of the *G. dulcis* reactions received confidence scores of 2, where only sequence data was available. Identified genes were manually assigned to the corresponding reactions in the Excel version of the model (File S1). Out of the annotated reactions, approximately 10% had confidence scores of 1, indicating no available evidence for those reactions (i.e., 125 reactions; File S1). These were either transport or gapfilled reactions, but were necessary for modeling and were only incorporated when there was supporting evidence for other reactions in the pathway. When available, Enzyme Commission (EC) codes and associated pathways were identified using KEGG and included in the Excel version of the GEM^38^.

Once the manual curation was complete, a FROG report was generated using FLUXER^39^, and the GEM was tested in MEMOTE^40^ to determine growth rate robustness using different simulators. MEMOTE also identified orphan and dead-end metabolites, and universally blocked reactions. These specific metabolites can only be consumed or produced by the model (i.e., for orphan or dead-ends, respectively). Universally blocked reactions are those that cannot carry a flux even without constraints imposed by the growth medium. Both orphan and dead-end metabolites and universally blocked reactions represent knowledge gaps that can be filled in the future with additional data^30^. These reactions and metabolites were documented in File S2.

The final curated model was deposited in the EMBL-EBI BioModels database and was designated with the perennial identifier: MODEL2303050001. The full FROG report, and model in SBML, YAML, MATLAB, JSON, and Excel formats can be found in File S1**,** and on GitHub (https://github.com/Drrachelmoore/Gloeocapsopsis_dulcis-GEM) where it will be curated with additional data in the future.

### Constraints and flux balance analysis (FBA)

We developed the GEM growth medium based on the BG-11 recipe used to grow *G. dulcis* in the laboratory (File S1) following the methods of Marinos et al.^41^. *iGd895* growth was then simulated with FBA in COBRApy (Version 0.25.0; Ebrahim et al.^29^) with the Gurobi Optimizer in Python (Version 3.10; Van Rossum et al.^42^) using the PyCharm IDE. Briefly, we used the model.optimize() function to run the FBA and determine optimal growth rates within the constraints of the BG-11 medium. We also simulated single reaction deletions using the knock_out() function to determine reaction essentiality for both water-uptake constrained and unconstrained simulations. Water exchange with the environment was restricted to 2 mmol gDW^−1^ day^−1^ in the constrained FBA simulations. This resulted in a maximum yield of 0.1 g H_2_O gDW^−1^ (i.e., 6 mmol H_2_O gDW^−1^), which roughly corresponded to an A_w_ of 0.5^6,43^.

### Comparison of metabolic states

We used the COMparison of METabolic states (ComMet, v1.0) method in MATLAB (version 9.13.0.2049777, R2022b) to compare metabolic states for *G. dulcis* with and without water constraints^44,45^. Default parameters were used unless stated otherwise. Briefly, the ComMet method works by creating a constrained and an unconstrained version of a GEM, where the constraint is placed on the uptake of a specific substrate. It then follows a seven-step pipeline to decompose the flux spaces into modules, conduct principal component analysis, basis rotation, and independent component analysis to compare the metabolic states. Specifically, we constrained the water uptake to 12 mmol gDW^−1^ h^−1^ for the constrained GEM to investigate changes in the *iGd895* metabolic network with xeric stress. Following ComMet preprocessing of the GEM flux spaces, we determined the flux distribution for all of the reactions in the unconstrained and constrained GEMs using the expectation propagation algorithm^46^. Next, we used ComMet’s PCA-based approach to decompose the flux spaces and extract global modules. Finally, xeric-stress-specific modules were identified using independent component analysis. Following analysis with ComMet, Cytoscape (Version 3.9.1) was utilized to plot the module networks^47^. R and R Studio (Version 2021.09.0) along with the ggplot2 library were used to generate plots of the flux distribution data^48,49^.

## Results and discussion

### *Gloeocapsopsis dulcis* genome

Genomic characteristics for *G. dulcis* are summarized in Table 1. Following sequence assembly in Trycycler^13^ we obtained a genome comprised of a ~ 5.29 Mbp chromosome and two plasmids, 11,484 and 307,228 bp in size. The mean read depth of the main chromosome was 492× as determined by Polypolish^20^. The mean read depth for the larger and smaller plasmid was 518× and 1040×, respectively. Following genome polishing with POLCA^21^, the consensus quality and consensus QV were estimated to be 99.9999 and 61.22, respectively, corresponding to an expected value of 4 errors across the genome. We then used the CheckM lineage workflow^22^ to determine a genome completeness of 100%, and a low potential sequence contamination of 1.44% (Fig. S1) (e.g., from cross-barcode reads). The predicted level of sequence contamination was lower than the recommended threshold (i.e., 5%) for classifying a genome as contaminated^50^.

**Table 1:** Comparison of *G. dulcis* characteristics between closed and draft sequence.

Study	This study	Puente-Sánchez et al.^51^
Genome status	Closed genome	Draft genome
Size	5,618,434	5,443,570
Contigs	3 (chromosome and two plasmids)	137
GC content (%)	42.5	42.42
Coding sequences (CDS)	5754	5641
RNAs	45	43

The overall genome included 5754 coding sequences and 45 RNA genes. The GC content was 42.5%. The general characteristics (e.g., size, GC content, etc.) of the closed genome matched well with the original draft sequence produced by Puente-Sánchez et al.^51^ (Table 1). One hundred and ten more coding sequences and two more RNA genes were identified in the closed genome as compared to the original draft.

**Figure 1:**
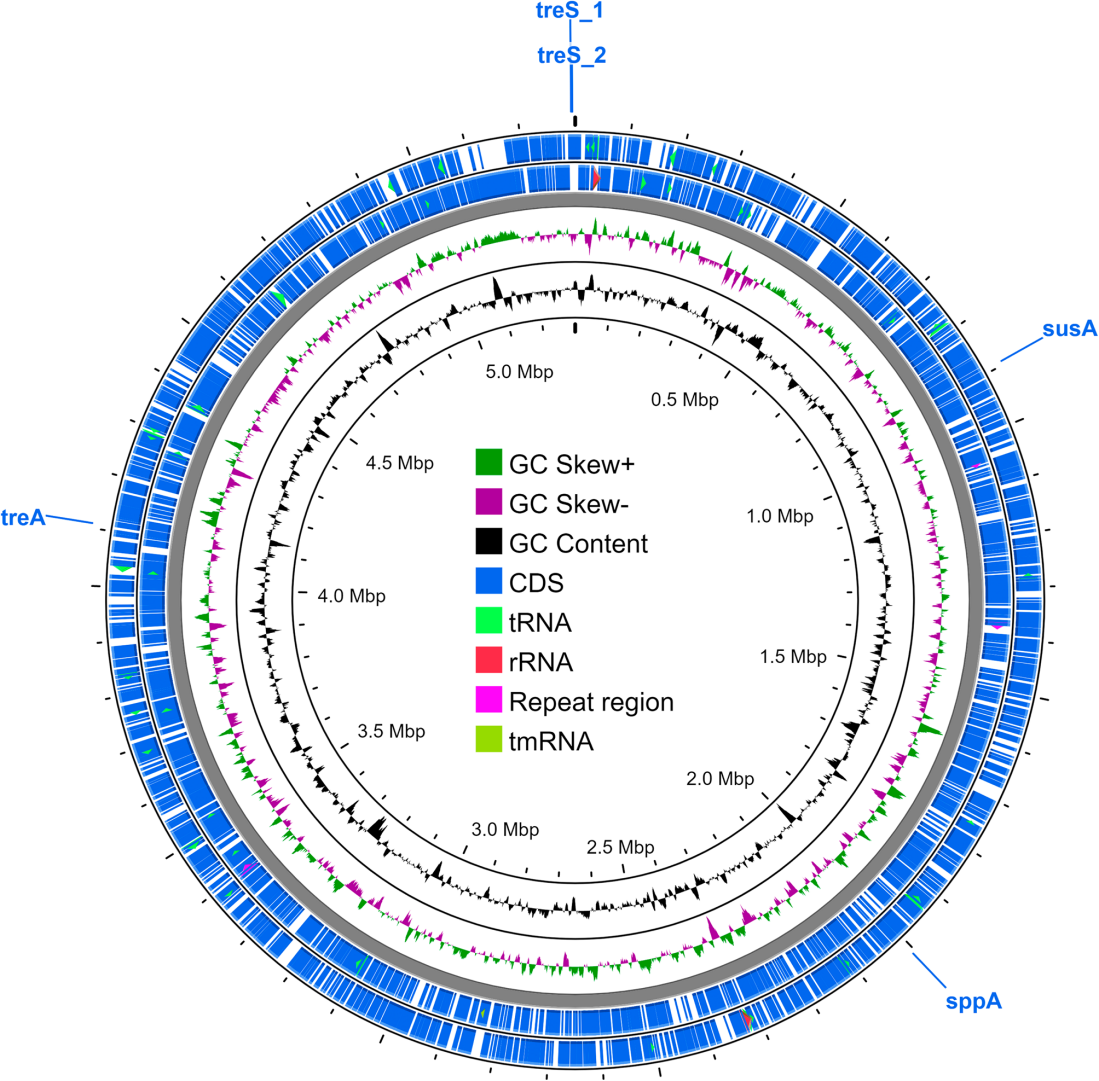
Map of the *G. dulcis* chromosome. Starting from the outer ring, rings one and two show coding sequences (CDS) from the forward and reverse strands, respectively. Green and red arrows within rings one and two depict tRNA and rRNA. Ring three depicts GC skew; ring four shows GC content. The gene names in refer to the following enzymes: sucrose synthase (i.e., *susA*; EC 2.4.1.13; gene 916), sucrose-phosphate phosphatase (i.e., *sppA*; EC 3.1.3.24; gene 3354), trehalose synthase (i.e., *treS_1*, *treS_2;* EC 5.4.99.16, gene 5376 and 5379), and trehalase (i.e., *treA*; EC 3.2.1.28; gene 4126).

The chromosome and plasmids were annotated and visualized with Proksee to further analyze the genome (Figs. 1 and S2). Azua-Bustos et al.^2^ previously identified two candidate genes involved in the synthesis of the compatible solute sucrose: sucrose 6-phosphate synthase and sucrose 6-phosphate phosphatase. We identified the location of those genes on the chromosome, as well as other potential genes associated with the production of trehalose, which is another compatible solute produced by *G. dulcis* (Fig. 1). We also identified other potential stress-related genes (supplemental results and discussion). These genes were discovered in both RAST and Prokka annotations, which were carried out separately, to confirm their presence and location in the genome^23,24^.

### Metabolic model reconstruction

GEMs enable the exploration of an organism’s metabolic capacities under various environmental conditions. We generated a new GEM (*iGd895*) using the *G. dulcis* genome to further elucidate the potential mechanisms underlying desiccation tolerance (File S1). *iGd895* consisted of 1251 reactions and 1198 metabolites (Table 2). Of the 1251 reactions, 98 were exchange reactions, and 74 were reactions that were automatically gapfilled using KBase on complete medium^26^. Exchange reactions are those that represented the nutritional boundaries of the system^30^. Gapfilled reactions are those that were added to the GEM to fill pathway gaps in the metabolic network.

**Table 2:** Characteristics of the reconstructed model of *G. dulcis*.

Features	This study
Total reactions	1251
Annotated reactions^a^	1147
Reactions with known genes^b^	1024
Exchange reactions	98
Demand reactions	6
Universally blocked reactions	418^c^
Gapfilled reactions	74
Metabolites	1198
Orphan metabolites	90
Dead-end metabolites	100

Exchange reactions are those that represent the system boundaries.

^a^Does not include exchange or demand reactions.

^b^Does not include pseudo-reactions like biomass production.

^c^Does not include blocked exchange reactions.

*iGd895* included three compartments, the cytosol, extracellular space, and the thylakoid, which is the membrane-bound compartment that accommodates photosynthetic machinery and reactions in cyanobacteria^52^. The total functional classification of the 1147 metabolic reactions (i.e., non-exchange reactions) is presented in Table S2. The complete list of all reactions is located in the model files in File S1.

### Validation of the FBA simulation using cultivation data

**Figure 2:**
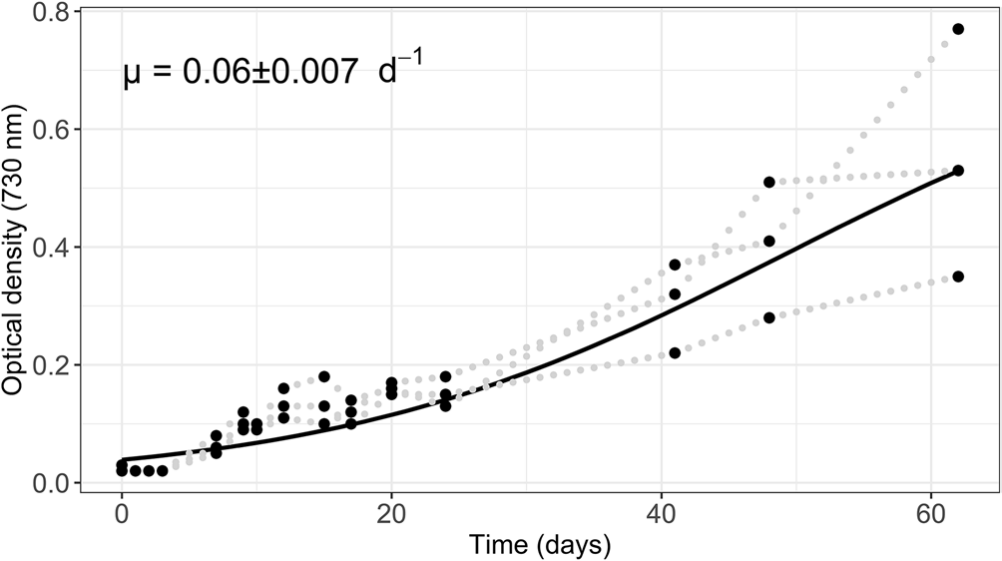
Growth curve and specific growth rate (µ) of *G. dulcis* grown in BG-11 liquid medium.

To verify the accuracy of *iGd895*, we compared the model’s growth rate with experimental growth rates. We found that an OD_730–750_ of 0.1 for *G. dulcis* corresponded to a cell count of 6.9 ± 0.45 × 10^5^ ml^−1^. We determined the in vitro growth rate of *G. dulcis* to be 0.06 ± 0.007 day^−1^ (Fig. 2), which is equivalent to the growth rate determined in Azua-Bustos et al.^2^ for *G. dulcis* grown at a light intensity of 10 μmol quanta m^−2^ s^−1^ (i.e., 0.0649 ± 0.0096 day^−1^; values extracted from figure).

The simulated specific growth rate of *iGd895* on the in-silico BG-11 growth medium was found to be 0.0694 day^−1^, falling within the range of the experimentally observed growth rates. This indicated that the model adequately replicated the metabolic activities that take place in *G. dulcis* on BG-11. Still, caution should be applied to the interpretation of *iGd895* results as further validation would require additional experimental data that was beyond the scope of this study and not yet available in the literature.

### In silico testing of compatible solute synthesis

We investigated the following reactions to determine which were involved in trehalose or sucrose production in *iGd895* under clement and desiccating conditions: reactions rxn01134 and rxn01966 for trehalose production, and rxn00577, rxn00578, and rxn00579 for sucrose production (supplemental results and discussion). In *iGd895*, trehalose was only produced under constrained water conditions, and only through reaction rxn01966. Trehalose was consumed, as expected, via reaction rxn00007 which encoded the enzyme trehalase. Trehalose was not produced or consumed in the unconstrained model. Notably, Azua-Bustos et al.^2^ did not detect trehalose via HPLC in *G. dulcis* cultures until one week after desiccation onset, suggesting its role as a specific response to extreme water stress.

Reaction rxn01966 encoded trehalose phosphorylase (i.e., EC 2.4.1.64, TreP), which catalyzes the reversible hydrolysis of trehalose to produce glucose-1-phosphate and glucose. While the TreP enzyme has been found in cyanobacteria (e.g., Murik et al.^53^), in vivo evidence of trehalose synthesis has only been shown with TreP isolated from fungi and certain bacteria like *Caldanaerobacter subterraneus* subsp. *Tengcongensis* and *C. subterraneus* subsp*. pacificus* (e.g., Ren et al.^54^, *C. subterraneus* was first reported as *Thermoanaerobacter tengcongensis*; Van der Borght et al.^55^). In constrained *iGd895*, rxn01966 (i.e., TreP) synthesized 0.0023 mmol gDW^−1^ day^−1^ of trehalose, which suggested that trehalose phosphorylase may be able to function in the synthesis direction for *G. dulcis*. It should be noted that although trehalose was exclusively produced by TreP in constrained *iGd895*, it can also be generated by trehalose synthase when it is set as the objective function (i.e., rxn01134). Thus, while we anticipate that TreP would be upregulated in desiccated *G. dulcis*, TreS may also be upregulated to synthesize trehalose in vivo.

### In silico analysis of knockouts and condition-dependent growth

We simulated reaction knockouts for *iGd895* to ascertain reaction essentiality for growth in conditions of unconstrained and constrained water uptake in BG-11 medium (File S4). In the constrained model, we limited water import via reaction rxn05319 to 2 mmol gDW^−1^ day^−1^. Anything lower than this value resulted in an infeasible solution. For this reason, we interpreted the knockout results as *G. dulcis* experiencing dehydration stress, but not yet fully desiccated, and that these results can be used to investigate potential desiccation-tolerance strategies.

Essential reactions were characterized as those that, when knocked out, resulted in no growth. Conversely, beneficial reactions were identified as those that resulted in an increased growth rate when knocked out. Nonessential and inessential reactions were defined as those that either had no impact on the growth rate or only had a minor impact (e.g., < 0.001 difference; supplemental results and discussion). After conducting knockout simulations, we found that both models had at least 395 essential reactions (Table 3). In the constrained model, two additional essential reactions (i.e., R0006 and R0039) were required for growth. R0039 represented the incident photon flux at 620 nm absorbed by the phycobilisomes, which are protein complexes anchored to the thylakoid membrane in cyanobacteria. These complexes can absorb light within the 600–650 nm range using the pigment phycocyanin^56^. R0006 represented the change in energy transfer, where light energy absorbed by the phycobilisome was delivered to photosystem II^31,57^.

**Table 3:** Comparison of essentiality results between unlimited and constrained model.

Reaction category	Unlimited model	Constrained model
Essential	395	397
Beneficial	0	1
Nonessential	861	848
Inessential	0	10
Total reactions	1251	1251

In *iGd895,* photosynthesis with constrained water was dependent on reaction R0006 to transfer energy to photosystem II, indicating that the phycobilisome supported growth in water limited conditions. The identification of R0039 as an essential reaction in the constrained *iGd895* model suggested a preference for the red-light spectrum when the objective is optimal growth. Cyanobacteria are known to concentrate their absorption efficiency within a narrow range of visible light wavelengths from 660 to 700 nm^58^. Cyanobacterial photosystems are less efficient at absorbing light in the green band of the electromagnetic spectrum, which typically ranges from 500 to 600 nm. This difference in absorption efficiency has been previously described by Gundlach et al.^59^ as the “Green Gap”.

### Comparison of unconstrained and constrained metabolic states

#### Applying constraints and the analytical approximation of fluxes

We used the ComMet pipeline^45^ to investigate the significant differences in reaction fluxes between the water-constrained and unconstrained metabolic states of *iGd895.* Unlike FBA, which relies on a specific objective function such as biomass production, the ComMet pipeline does not require such a constraint. This allowed us to more effectively consider the desiccation-tolerance mechanisms of *G. dulcis*, which are unlikely to involve the optimization of growth. Similar to the above FBA-based analysis, we first generated two versions of the *iGd895*: ‘unconstrained’ and ‘constrained’. In the constrained model, the bounds for water uptake were limited to 12 mmol gDW^−1^ day^−1^, as opposed to an unconstrained flux at 1000 mmol gDW^−1^ day^−1^. Lower bounds for water uptake (e.g., 0–11 mmol gDW^−1^ day^−1^) prevented the expectation propagation algorithm from converging. Thus, as before, we did not consider it a true ‘desiccated’ model.

Further preprocessing was carried out following the ComMet pipeline to remove 900 blocked reactions (i.e., those that were unable to carry a flux under the imposed conditions) from each model. Apart from the water constraint, ComMet set the bounds for the other exchange reactions in the models to allow for unlimited nutrient uptake, effectively removing them as a factor. However, to simulate photoautotrophy, 70 exchange reactions were removed from both models (edited ComMet code on GitHub). For example, exchange reactions of amino acids, nitrate, and organic carbon sources were removed to block mixotrophic metabolism and to better represent experimental lab testing scenarios. This preprocessing narrowed the unconstrained and constrained GEM to 289 and 282 reactions, respectively, from the original set of 1251.

**Figure 3:**
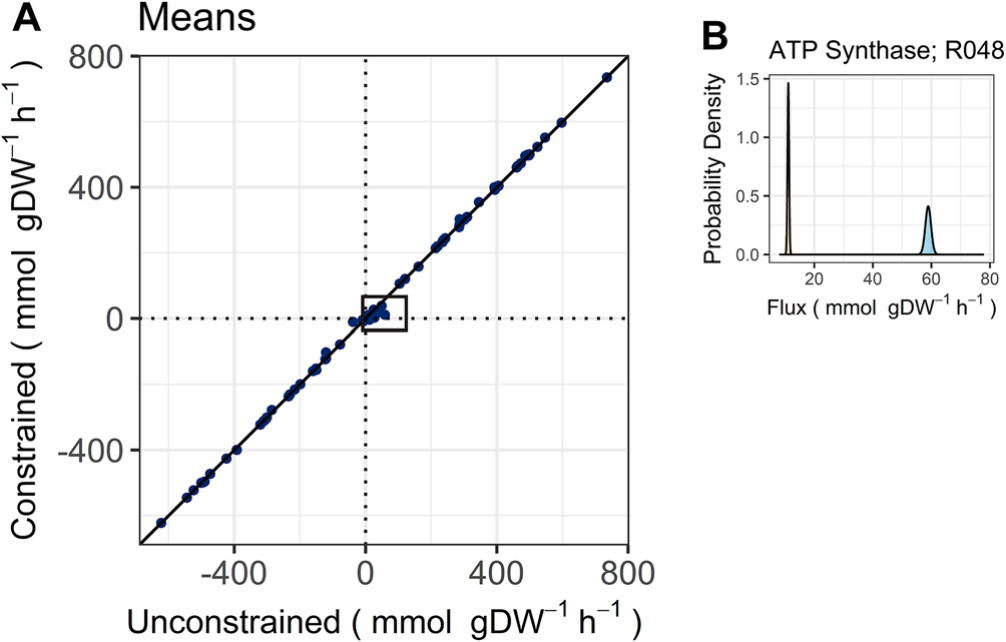
Comparison of the means (**A**) of individual reactions between the constrained and unconstrained model simulations. The black box highlights the reactions that were impacted by water limitation. Flux distributions are shown for one of the reactions significantly affected (p << 0.05) by water limitation (**B**), the thylakoid-based ATP synthase reaction. Its flux distributions are shown for both the unconstrained (blue) and constrained (orange) simulations.

Following preprocessing, we used the expectation propagation algorithm from Braunstein et al.^46^ in ComMet to approximate the feasible spaces of both GEMs, and in doing so, calculated the mean and standard deviations of the flux distribution for every reaction in the simulations. We produced a reaction-wise comparison plot of the means (Fig. 3A), as described in Sarathy et al.^45^ to visually inspect the differences that resulted from constrained water. Overall, we found that a majority of the reaction means were highly regulated and exhibited comparable summary statistics between the two metabolic states. However, some of the reactions displayed a noticeable deviation from the identity line, which indicated a change in the flux (Fig. 3A). Our analysis revealed that 26 of these reactions, not including those that were blocked with constrained water, exhibited significant changes in their flux distribution between the conditions (File S5, p << 0.05). Notably, these reactions were involved in several key metabolic pathways including photosynthesis, glycolysis, and the pentose phosphate pathway.

**Figure 4:**
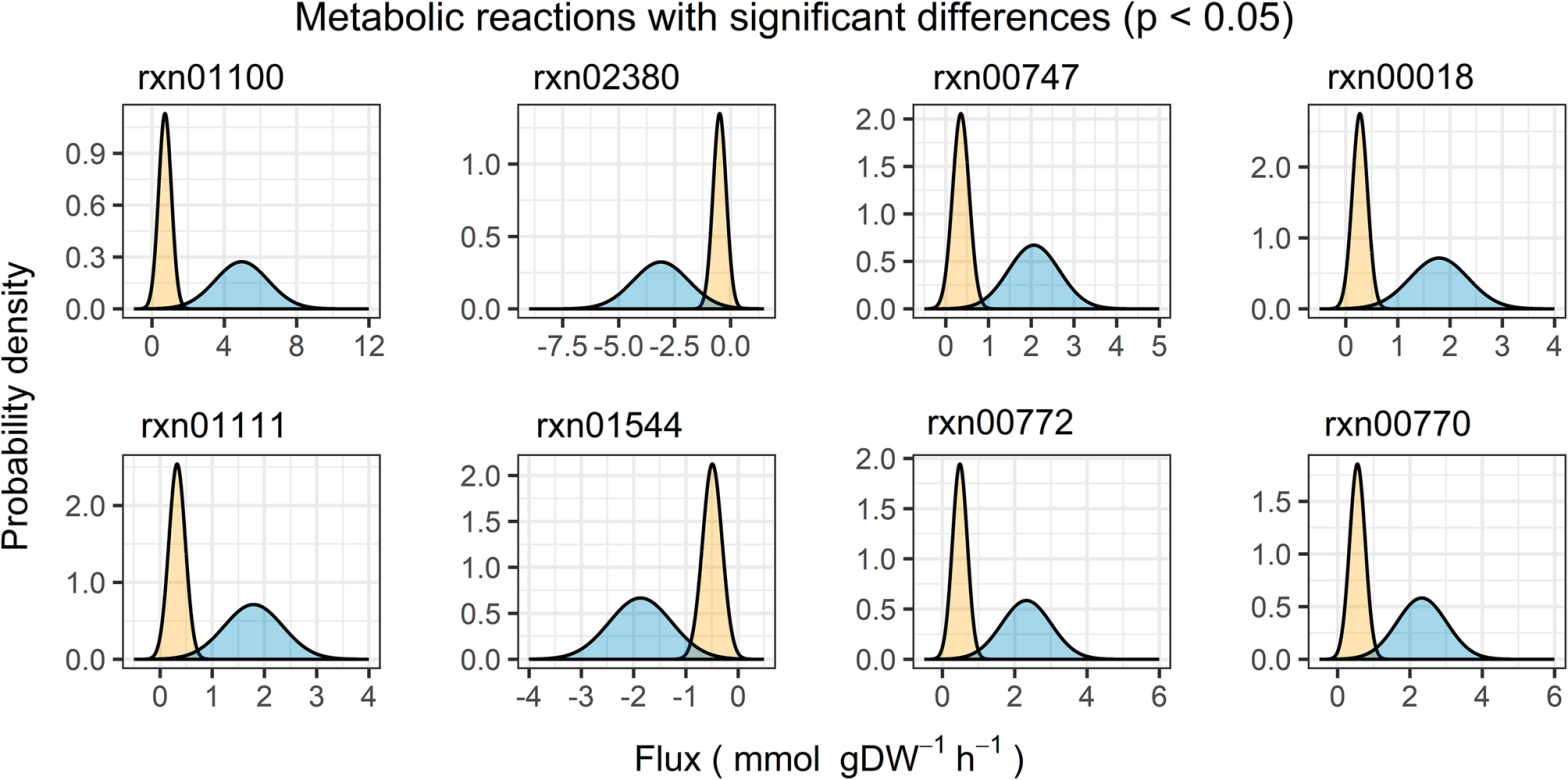
Flux distributions for reactions within the glycolysis (rxn01100, rxn02380, rxn00747), carbon fixation in photosynthetic organisms (rxn00018, rxn01111), purine metabolism (rxn01544), and pentose phosphate (rxn00772, rxn00770) pathways with significant differences in flux statistics between unconstrained (blue) and constrained (orange) simulations.

We created density plots to visualize the significant changes in the flux distributions (Figs. 3B and 4). The flux distributions for the water-constrained simulation exhibited notable reductions compared to those in the unconstrained simulation. These reductions were reflected in both the location and shape of the distributions between the two simulations. More specifically, we observed that negative flux distributions shifted toward the right, and positive distributions shifted toward the left of the unconstrained simulation. The observed shifts in flux distribution suggested that the reactions may have been either downregulated or shifted toward a near-zero flux state, which could occur in vivo as a mechanism to reduce the occurrence of ROS-producing reactions and oxidative stress. The shifts in flux distribution were especially apparent for the ATP synthase and photosynthetic reactions (Fig. 3B, File S5). Furthermore, the overall shape of the distributions changed from broadly dispersed in the unconstrained simulation to relatively narrower ranges, indicating a tighter regulation of the reaction fluxes (Fig. 4).

These results are consistent with the current understanding of how certain cyanobacteria respond to desiccation in that several energy-consuming pathways tend to be downregulated. For example, Katoh et al.^60^ demonstrated that genes related to photosynthesis, ATP synthase, and carbon fixation were downregulated in the cyanobacterium *Anabaena* sp. PCC7120 following desiccation stress. Notably, downregulation of photosynthesis-related genes has also been observed in green algae in response to desiccation, and has been thought to reflect a shift from growth to energy-saving metabolism^61^.

Studies have also demonstrated that certain proteins involved in glycolysis exhibit reduced expression levels during cellular dehydration in cyanobacteria. In particular, the gene for phosphoglycerate kinase (*PGK*, EC 2.7.2.3) was found to be downregulated and the enzymatic activity of triose-phosphate isomerase (TPI, EC 5.3.1.1) was found to decrease in response to water loss in *Nostoc flagelliforme*^62,63^. Consistently, our model revealed significant decreases in reaction flux for these glycolytic enzymes represented by reaction rxn01100 and rxn00747 in *iGd895* when water uptake was constrained (Fig. 4). Further, reaction rxn02380, which is catalyzed by glucose-6-phosphate isomerase (PGI, EC 5.3.1.9), shifted toward a near-zero flux state with water constraints. This shift indicated the potential for the reaction to reverse direction from converting beta-D-fructofuranose 6-phosphate to alpha-D-glucose 6-phosphate. *PGI* expression has been seen to increase in *N. flagelliforme* with water stress, potentially as a mechanism to enhance the synthesis of polysaccharides like sucrose^64^.

Reaction rxn00018 (Fig. 4), catalyzed by ribulose-bisphosphate (RuBisCO, EC 4.1.1.39), has also been shown to decline in activity in water-stressed higher plants^65^ and cyanobacteria^66^. Similarly, it was also downregulated in dehydrated *Anabaena* sp. PCC 7120^67^, suggesting that carbon fixation is affected by water stress in cyanobacteria. Further, the significant reduction in RuBisCO and phosphoribulokinase (rxn01111, EC 2.7.1.19) activity could contribute to the modulation of carbon fixation and the allocation of carbon resources to glycogen storage^68^. Reduction of pentose phosphate pathway reaction fluxes could also help to conserve resources that could then be redirected toward glycogen synthesis. Reactions rxn00772 and rxn00770 (Fig. 4), corresponding to pentose phosphate pathway enzymes ribokinase and ribose-phosphate diphosphokinase (i.e., EC 2.7.1.15 and 2.7.6.1, respectively), were reduced significantly with respect to the unconstrained reaction fluxes. This may also limit oxidative stress through the reduction of ROS production.

#### Identification of xeric stress-specific modules

As demonstrated above, water limitation significantly affected the metabolic network in several key pathways. To further elucidate the interplay between reactions across the entire network, we examined “modules”, or groups of interacting reactions, whose flux variability accounted for the total variation of the simulation flux space. In other words, we used the ComMet approach^45^ to first identify “global modules” (supplemental results and discussion), which we then utilized to uncover unique patterns of metabolic regulation between the unconstrained and constrained simulations using independent component analysis (ICA).

**Figure 5:**
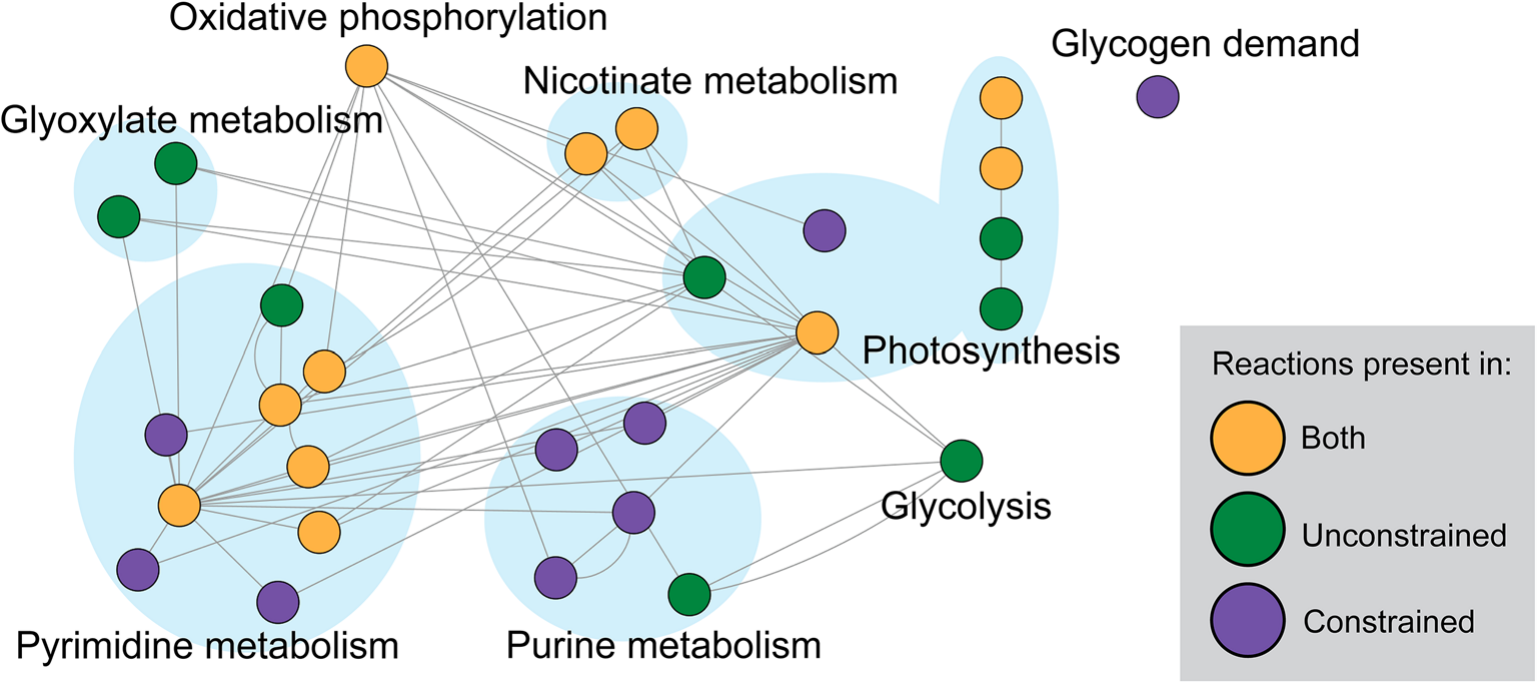
Combined network of distinct modules extracted from the 82 PCs that were biochemically distinct between the unconstrained and constrained reactions. Reactions are grouped by KEGG pathway. Pathways with more than one reaction present are highlighted with the blue underlay. Node color indicates the condition: constrained (purple), unconstrained (green), and both (orange). Edges indicate shared metabolites. The network is available on NDEx (https://doi.org/10.18119/N9QK6F).

ICA was performed on a combined set of 82 principal components (PCs; 41 from each condition). These PCs were chosen as they accounted for 99.9% of the flux space variation in both simulations (supplemental results and discussion). Initially, the ICA optimization script from ComMet was executed using a bootstrapping approach to determine the optimal number of independent components required for decomposing the original multivariate signal, which was found to be 38 (Fig. S5). Subsequently, the combined set of rotated PCs from both simulations and the optimum number were utilized to perform the ICA for 9000 iterations. The ICA revealed distinct features that corresponded to 17 rotated PCs, and comprised a total of 28 reactions. Eleven of these reactions were part of both distinct modules from the constrained and unconstrained simulations (File S9), including essential reactions such as those involved in oxidative phosphorylation, photosynthesis, pyrimidine and purine metabolism, and nicotinate and nicotinamide metabolism. The ICA results are illustrated in Fig. 5 as a combined network/reaction map.

Only eight reactions were exclusive to the unconstrained network, while nine were exclusive to the constrained network, highlighting several relevant changes between the two metabolic states of the *G. dulcis* network (Fig. 5). Reactions belonging to the glyoxylate and dicarboxylate metabolism pathway (i.e., rxn01281 and rxn01280) and the glycolysis pathway (i.e., rxn00411) were exclusive to the unconstrained simulation. This indicated an emphasis on energy production to support growth and carbon fixation via the Calvin cycle when water was plentiful (Fig. 5). Rxn01281 and rxn01280 represented the reactions catalyzed by (R)-Glycerate:NADP+ oxidoreductase (GOR) and (S)-Glycerate:NAD+ oxidoreductase (SGOR) (EC 1.1.1.60). These enzymes are involved in the photorespiratory pathway, which operates in parallel with the carbon fixation pathway (e.g., the Calvin cycle) in photosynthetic organisms.

The photorespiratory pathway is responsible for the detoxification of glycolate, a toxic byproduct of the oxygenation reaction catalyzed by RuBisCO, and the recycling of carbon and nitrogen. Glycolate is converted to glyoxylate via the action of glycolate oxidase and then converted to glycerate by the action of GOR or SGOR, depending on the availability of NADP+ or NAD+. Glycerate can be further converted to 3-phosphoglycerate by the action of (R)-Glycerate kinase and used in the Calvin cycle for carbon fixation, thus contributing indirectly to carbon fixation. However, the absence of these two reactions (i.e., rxn01281 and rxn01280) in the constrained simulation, raises the possibility that *G. dulcis* could adjust its carbon fixation strategy via these pathways, especially when faced with limited water availability. This adjustment might encompass recycling or even excretion strategies, possibly as a response to stress. This dynamic response could significantly influence carbon management within the cell.

Notably, the reaction that represented the accumulation of glycogen (i.e., the glycogen demand reaction) was only present in the constrained simulation as shown in Fig. 5. This suggested that *G. dulcis* may use glycogen as a carbon-storage mechanism during water-limited conditions, similar to what was indicated in the flux distribution data above (e.g., Fig. 5). Glycogen can serve as a source of carbon and energy during extreme desiccation or darkness^69^ or as a metabolic sink for compatible solutes, allowing for the rapid modulation of intracellular osmolarity. Baran et al.^70^ proposed that cyanobacteria may respond to changes in salinity by converting glycogen into compatible solutes, rather than synthesizing them de novo, and this mechanism may also be relevant for desiccation tolerance.

Reaction rxn00411 was exclusively present in the unconstrained simulation (Fig. 5) and represented the final step of glycolysis, wherein pyruvate kinase (EC 2.7.1.40) converts phosphoenolpyruvate and ADP into pyruvate and ATP. Although photosynthesis is the primary means of generating ATP in photosynthetic organisms, cyanobacteria may utilize glycolysis as an additional pathway to produce ATP. The exclusive presence of this reaction in the unconstrained simulation indicated that the limited availability of water in the constrained model did not support the costs of glycolysis reactions.

ATP synthase (EC 7.1.2.2; reaction R048), which catalyzes oxidative phosphorylation, and ATP:NAD+ 2′-phosphotransferase (EC 2.7.1.23; rxn00077), which can catalyze the production of ATP from ADP and NADP+ in the nicotinate and nicotinamide metabolism pathway, were present in both simulations. However, there were significant differences in the mean fluxes (File S9) of reaction R048 between the two simulations, as shown by the flux distributions (Fig. 3B). The reduced flux via ATP synthase and the absence of ATP production through glycolysis (i.e., rxn00411) in the constrained simulation suggested that *G. dulcis* may prioritize energy conservation and/or employ the nucleoside-diphosphate kinase (EC 2.7.4.6) enzyme, in addition to the other two enzymes, in order to meet energy demands.

The presence of the nucleoside-diphosphate kinase-catalyzed reaction (i.e., rxn00237; purine metabolism pathway), which can generate ATP in cyanobacteria by transferring a phosphate group from a nucleoside triphosphate to ADP, was exclusive to the constrained simulation. This reaction may be preferred over the ATP synthase-catalyzed reaction as it requires less water for catalysis. Additional reactions exclusive to the constrained simulation in the purine (i.e., rxn01354, rxn00304, and rxn00840) and pyrimidine metabolism (i.e., rxn01128, rxn01129, and rxn00712) pathways may support this function (File S9). The latter reactions are involved the synthesis of dATP, whereas the former involved enzymes that can transfer a phosphate group to nucleoside diphosphate to form nucleoside triphosphates (e.g., dGTP, dATP, or GTP), all of which can be used in ATP production through the nucleoside-diphosphate kinase. Conversely, the only purine and pyrimidine metabolism reactions exclusive to the unconstrained simulation consume ATP to produce CTP and dCTP, respectively. This further indicated that *G. dulcis* may prioritize cellular processes such as DNA replication, RNA transcription, and growth primarily when water is abundant, but shift to prioritize energy production and conservation during water scarcity.

Photosynthesis reactions (R543, R0048, R0047) were present in both simulations, with R0047 and R0048 responsible for carotenoid transport and exchange, respectively. R543 represents the photosynthetic NADPH synthesis flux using ferredoxins. Only the unconstrained simulation contained reactions R0045 and R0046, which represent green light (i.e., 550 nm photon) exchange with the environment and absorption by the orange carotenoid protein. The only photosynthesis-related reaction exclusive to the constrained simulation was R0037, which represents quinol oxidase (EC 7.1.1.7), an enzyme that catalyzes the transfer of electrons from quinol molecules to molecular oxygen, producing water in the process.

The presence of quinol oxidase in the constrained simulation indicated that the production of metabolic water may be critical for survival during desiccation. For example, metabolic water production has been thought to sustain the hydration needs of cold desert soil-dwelling microbes^71^. The production of metabolic water may also play a role in supporting the stability of cellular structures, such as proteins and membranes, which are prone to damage and denaturation under desiccation stress. In addition, quinol oxidase is involved in the regulation of electron transfer in the respiratory chain and redox homeostasis, which may be crucial for maintaining cellular energy balance under stress conditions. Overall, the identification of quinol oxidase in the constrained simulation suggested that this enzyme could play a crucial role in generating metabolic water and maintaining redox balance, enabling survival in desiccating environments like the Atacama Desert.

## Conclusions

Our findings suggest that *G. dulcis* cyanobacteria exhibit a multifaceted metabolic response to desiccation. Specifically, our genome-scale modeling analysis revealed significant metabolic shifts that may occur during desiccation, particularly during the initial limitation of water. These shifts involved the reconfiguration of metabolism from ATP production/consumption to a greater emphasis on ATP conservation, as well as a reduced emphasis on carbon fixation in favor of glycogen accumulation. Furthermore, our results highlight the potentially critical role of metabolic water production for *G. dulcis* survival during desiccation. The significant changes in reaction fluxes between metabolic states imply a tight regulation of specific pathways under water limitation. Finally, the closing and annotation of the *G. dulcis* genome led to the identification of several stress response genes that are likely to be upregulated during desiccation. Collectively, these findings advance our understanding of how microorganisms may survive extremely arid environments.

### Supplementary Information

Supplementary File S1.

Supplementary File S2.

Supplementary File S3.

Supplementary File S4.

Supplementary File S5.

Supplementary File S6.

Supplementary File S7.

Supplementary File S8.

Supplementary File S9.

Supplementary Figures.

Supplemental Files and Tables: Table of Contents.

Supplemental Results and Discussion.

Supplementary Table S1.

Supplementary Table S2.

## Data Availability

The data supporting the findings of this study are available within the supplementary materials. Models are available in the supplementary materials, GitHub (https://github.com/Drrachelmoore/Gloeocapsopsis_dulcis-GEM), and the EMBL-EBI BioModels database (MODEL2303050001). Network reaction maps are available on NDEx (https://doi.org/10.18119/N9QK6F; https://doi.org/10.18119/N9V89Q, https://doi.org/10.18119/N9003X). The *G. dulcis* genome is available on NCBI (PRJNA941297; TaxID: 1433147).
