# Author Correction: Unveiling metabolic pathways involved in the extreme desiccation tolerance of an Atacama cyanobacterium

**DOI:** 10.1038/s41598-023-44829-6

**Published:** 2023-10-17

**Authors:** Rachel A. Moore, Armando Azua-Bustos, Carlos González-Silva, Christopher E. Carr

**Affiliations:** 1 School of Earth and Atmospheric Sciences, Georgia Institute of Technology, 275 Ferst Dr. NW, Atlanta, GA 30332 USA; 2 Centro de Astrobiología (CSIC-INTA), Madrid, Spain; 3 Instituto de Ciencias Biomédicas, Facultad de Ciencias de la Salud, Universidad Autónoma de Chile, Santiago, Chile; 4 Facultad de Ciencias, Universidad de Tarapacá, Arica, Chile; 5 Daniel Guggenheim School of Aerospace Engineering, Georgia Institute of Technology, Atlanta, GA 30332 USA

Correction to: *Scientific Reports* https://doi.org/10.1038/s41598-023-41879-8, published online 22 September 2023

The Acknowledgements section in the original version of this Article was incomplete.

“This work was supported by NASA Exobiology awards 80NSSC19K0469 (MIT) and 80NSSC22K0189 (Georgia Tech) to CEC, and the Human Frontiers Science Program grant RGY0066/2018 to AAB.”

now reads:

“This work was supported by NASA Exobiology awards 80NSSC19K0469 (MIT) and 80NSSC22K0189 (Georgia Tech) to CEC, and the Human Frontiers Science Program grant RGY0066/2018 and the European Research Council Grant no 818602 to AAB.”

The original Article has been corrected.

