# Supplementary material for: Unveiling metabolic pathways involved in the extreme desiccation tolerance of an Atacama cyanobacterium
Source: Sci Rep. 2023 Sep 22;13:15767. doi: 10.1038/s41598-023-41879-8 (PMC10516996; doi:10.1038/s41598-023-41879-8)
Supplement: Supplementary file 3 — Supplementary File S3. [file 41598_2023_41879_MOESM3_ESM.pdf]

RefSeq	chr1
OriC length	738 nt
OriC AT content	0.69
The location of oriC region	4134960..4135697 nt
The extremes of GC disparity	4933781 nt (minimum), 292325 nt (maximum)
OriC map	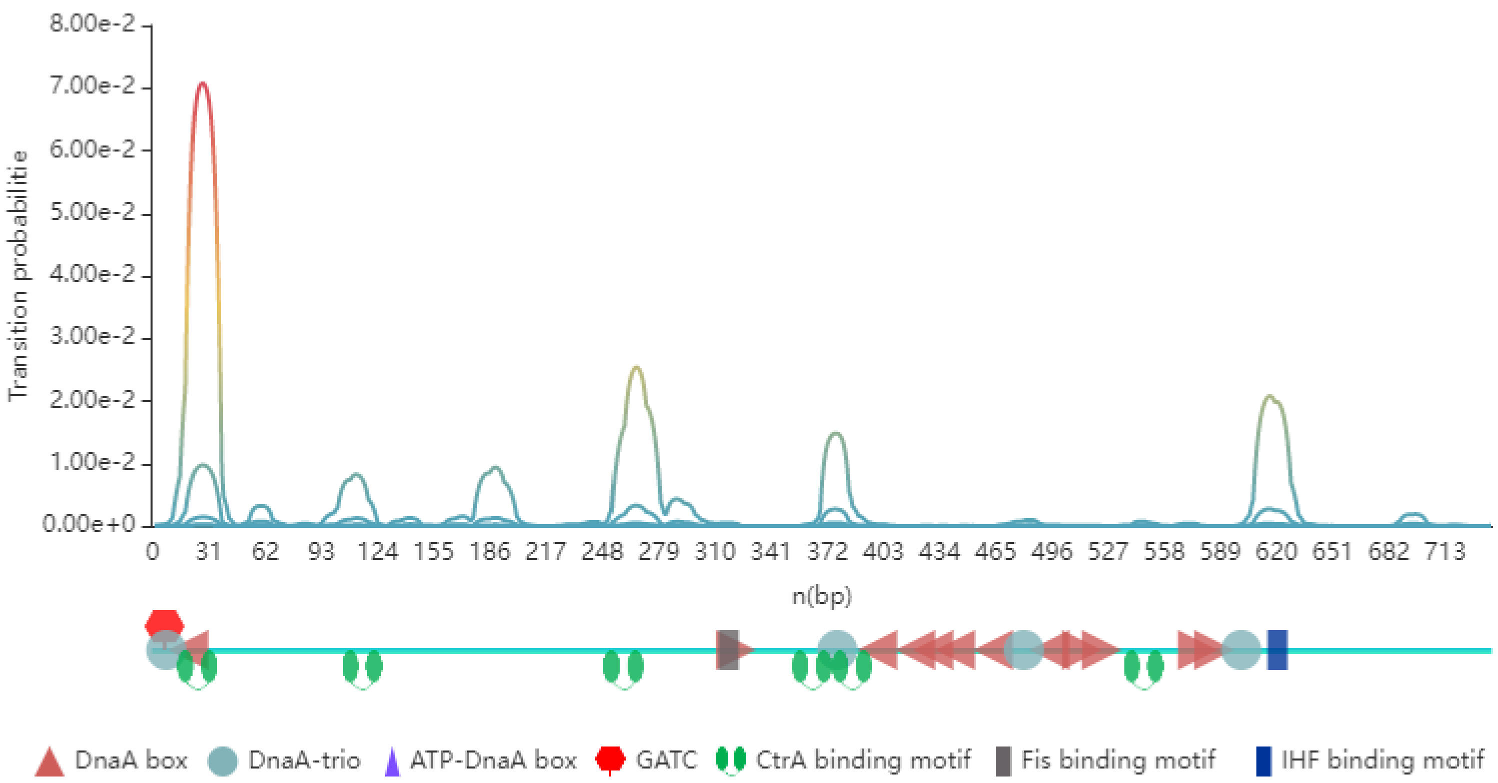
