# Supplementary material for: Unveiling metabolic pathways involved in the extreme desiccation tolerance of an Atacama cyanobacterium
Source: Sci Rep. 2023 Sep 22;13:15767. doi: 10.1038/s41598-023-41879-8 (PMC10516996; doi:10.1038/s41598-023-41879-8)
Supplement: Supplementary file 10 — Supplementary Figures. [file 41598_2023_41879_MOESM10_ESM.zip › Supplemental Figures/Supplemental figure legends.docx]

**Supplemental figure legends**

**Fig. S1** Visual representation of CheckM determined completeness, contamination, and strain heterogeneity within *G. dulcis* genome. Green bars represent marker genes that were present once, grey bars represent missing markers. Marker genes that occur more than once are indicated by blue, or orange and red bars, depending on amino acid identity. Amino acid identity ≥90% indicates heterogeneity, whereas anything lower indicates potential contamination.

**Fig. S2** Maps of the *G. dulcis* plasmids. Rings one and two show coding sequences (CDS) from the forward and reverse strands, respectively.

**Fig. S3** Cumulative variance (%) explained by the PCs of the flux spaces as determined for unconstrained and constrained simulations.

**Fig. S4** Network of the global modules from the unconstrained (top) and constrained (bottom) simulations. Edges represent shared metabolites, and nodes represent reactions. The color of each node corresponds to the number of PCs to which it belongs, with darker colors indicating a higher number of PCs. For interactive viewing of these networks, NDEx links are provided at <https://doi.org/10.18119/N9003X> and <https://doi.org/10.18119/N9V89Q> for the unconstrained and constrained networks, respectively.

**Fig. S5** ComMet ICA optimization for selecting the optimal number of features. The stability profiles of the bootstrapped Independent Component Analysis (ICA) runs are indicated by the grey lines, while the blue and red dashed lines represent the results of two-line clustering. The optimal number of independent components was determined as the point of intersection between the two lines and is indicated by the black vertical line.
