# Supplementary figures and images for: Unveiling metabolic pathways involved in the extreme desiccation tolerance of an Atacama cyanobacterium
Source: Sci Rep. 2023 Sep 22;13:15767. doi: 10.1038/s41598-023-41879-8 (PMC10516996; doi:10.1038/s41598-023-41879-8)

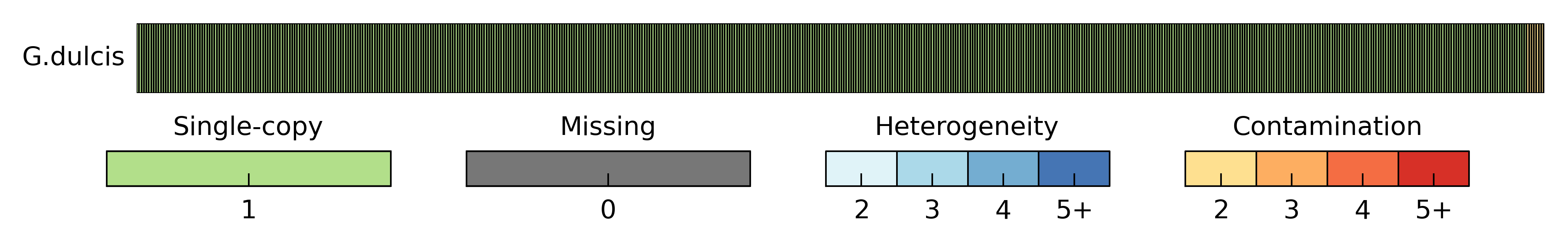

Supplement: Supplementary file 10 — Supplementary Figures. [file 41598_2023_41879_MOESM10_ESM.zip › Supplemental Figures/Figure S1.png]

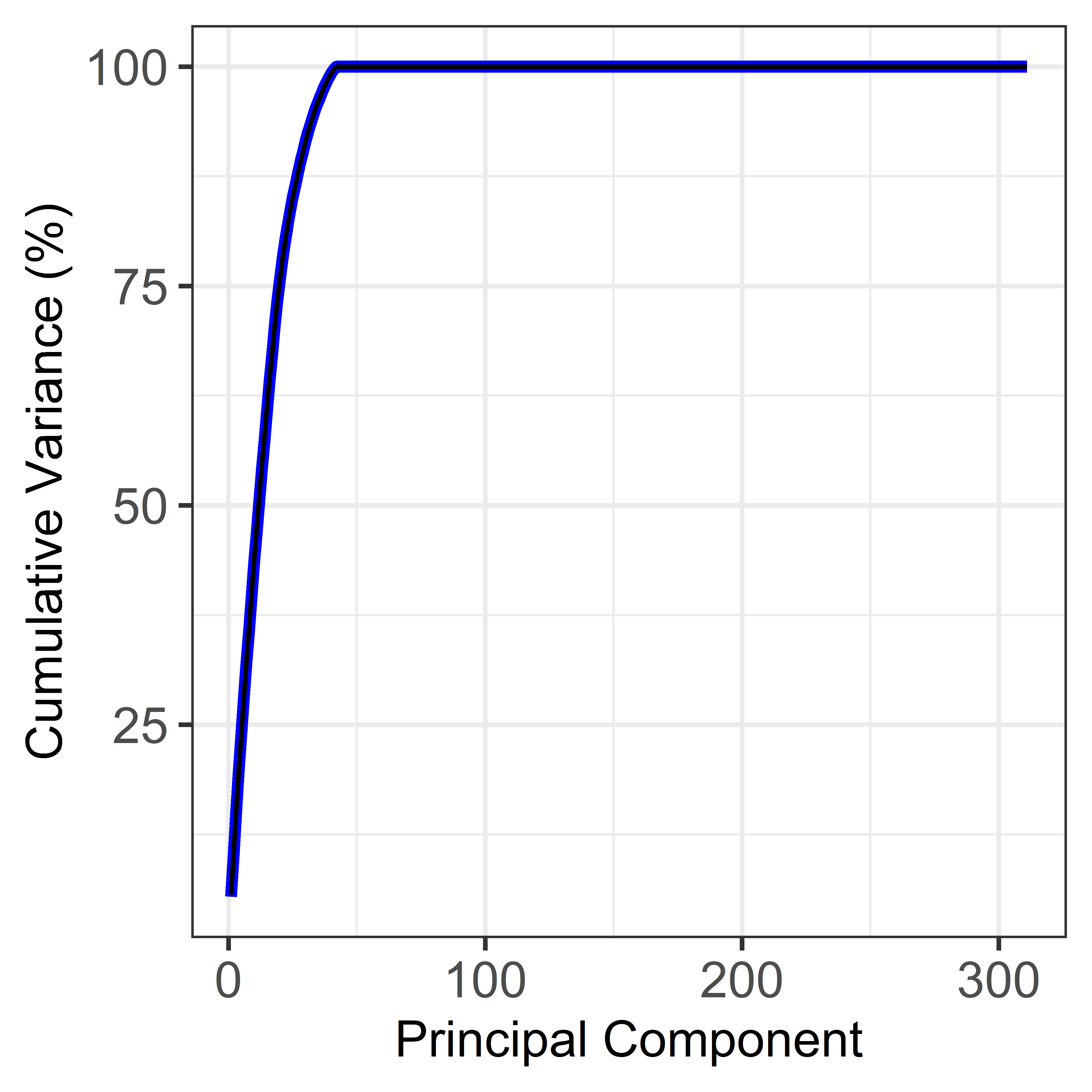

Supplement: Supplementary file 10 — Supplementary Figures. [file 41598_2023_41879_MOESM10_ESM.zip › Supplemental Figures/Figure S3.tiff]

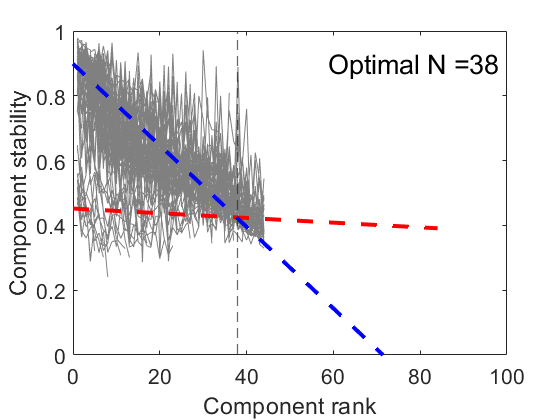

Supplement: Supplementary file 10 — Supplementary Figures. [file 41598_2023_41879_MOESM10_ESM.zip › Supplemental Figures/Figure S5.png]
