# Supplementary material for: Unveiling metabolic pathways involved in the extreme desiccation tolerance of an Atacama cyanobacterium
Source: Sci Rep. 2023 Sep 22;13:15767. doi: 10.1038/s41598-023-41879-8 (PMC10516996; doi:10.1038/s41598-023-41879-8)
Supplement: Supplementary file 11 — Supplemental Files and Tables: Table of Contents. [file 41598_2023_41879_MOESM11_ESM.docx]

**Supplemental Files and Tables**

***File S1***

-BG-11 media recipe and in silico breakdown

-*G. dulcis* JSON model

-*G. dulcis* MATLAB model

-*G. dulcis* EXCEL model

-*G. dulcis* XML model

-*G. dulcis* YAML model

-*G. dulcis* RAST annotated genome

***File S2***

-List of universally blocked reactions, orphan metabolites, and dead-end metabolites

***File S3***

-Data from Ori-Finder (i.e., most probable origin of replication on chromosome)

***File S4***

-Growth rate with single reaction knock outs in constrained or unconstrained model. Very small numbers are equivalent to 0. Empty cells indicate infeasibility.

***File S5***

-Reaction-wise summary statistics computed using analytical approximation of fluxes in ComMet.

***File S6***

-Variance explained by individual and cumulative numbers of PCs for the constrained (con) and unconstrained (un) simulations.

***File S7***

-Global module for unconstrained simulation, organized by PC number, shared name (i.e., reaction), and pathway.

***File S8***

-Global module for constrained simulation, organized by PC number, shared name (i.e., reaction), and pathway.

***File S9***

-Distinct network modules, organized by reaction ID (con = constrained, un = unconstrained); mean = mean flux for simulation, numPCs = number of PCs reaction was involved in, std = standard deviation.

***Table S1***

-Initiator recognition sequences in G. dulcis as predicted by Ori-Finder

***Table S2***

-The total functional classification of the metabolic reactions
