# Supplementary material for: Unveiling metabolic pathways involved in the extreme desiccation tolerance of an Atacama cyanobacterium
Source: Sci Rep. 2023 Sep 22;13:15767. doi: 10.1038/s41598-023-41879-8 (PMC10516996; doi:10.1038/s41598-023-41879-8)
Supplement: Supplementary file 12 — Supplemental Results and Discussion. [file 41598_2023_41879_MOESM12_ESM.docx]

**Supplemental Results and Discussion**

*Gloeocapsopsis dulcis* *genome oriC*

We used Ori-Finder 2022 ^1^, which analyzes the GC skew, *oriC* functional elements and the location of indicator genes (e.g., *dnaA*, *dnaN*, *hemB*, etc.), to identify the origin of replication (e.g., predicted DnaA boxes in **Table S1**). Ori-Finder predicted two potential *oriC* regions, however, based on homology with other *oriC* sequences and GC skew, the most likely replication origin started at 4,134,960 nt within the chromosome sequence (**File S3**).

*Gloeocapsopsis dulcis* *stress-related genes*

Cyanobacteria express a number of enzymes to cope with desiccation stress in addition to producing compatible solutes. For example, we identified two possible genes for manganese catalases at different locations in the genome (EC 1.11.1.6) (**File 1**). Catalases are antioxidant enzymes that prevent oxidative cell damage by converting H_2_O_2_ to water and oxygen. Reactive oxygen species, like H_2_O_2_, accumulate in desiccating bacterial cells ^3^. In particular, manganese catalases use Mn atoms in their active site ^52^. Manganese catalases (e.g., KatB) have been shown to be expressed under desiccation stress in the cyanobacterium *Anabaena* ^53^. Further, a mutation to the manganese catalase reduced the viability of *Anabaena* PCC 7120 in response to desiccation ^54^.

In the genome, we identified several potential genes for heat shock and stress-related proteins like GroEL (i.e., gene 2146 and 3605), GroES (i.e., gene 3604), DnaK (i.e., gene 2258, 4326, and 4843), DnaJ (i.e., gene 408), and GrpE (i.e., gene 2273 and 2274), and universal stress protein Sll1388 (i.e., gene 3249 and 5130). Heat shock proteins often function as chaperones for protein folding and establishment of proper conformation. For example, GroEL forms a ~900 kDa cylinder, that when in conjugation with GroES (i.e., a dome-like “lid” protein), creates a folding chamber for proteins ^55^. DnaK is a ~70 kDa protein that also mediates protein folding in conjunction with other proteins, namely, DnaJ and GrpE ^56^. Sll1388 is a universal stress protein that is thought to protect the cell from stress as it is expressed in response to toxic concentrations of H_2_O_2_ in the cyanobacterium *Synechocystis* sp. PCC 6803, however its exact mechanism is unknown ^57^. Similar to what has been seen in other cyanobacteria (e.g., ^53^), we anticipate that these stress-related genes and catalases may be up-regulated in response to desiccation stress in *G. dulcis*.

*Metabolic Model Reconstruction*

We found that several reactions in *iGd895* were universally blocked, meaning that they could not carry a flux under any condition (**Table 2**). However, genes representing their associated enzymes were located in the genome for all but 13 of those reactions (**File S2**). Similarly, we identified several orphan and dead-end metabolites (**Table 2**), which were only consumed but not produced or are only produced by not consumed by reactions in the model, respectively. However, the corresponding reactions for all of the metabolites except four of the dead-end metabolites had known genes associated with them (**File S2**). The existence of these blocked reactions and orphan or dead-end metabolites in *iGd895* indicated knowledge gaps, as genomic data suggested their potential utilization, yet the mechanisms to connect them with other reactions and enable flux remains unknown. For this reason, none of these reactions or metabolites were removed from the model, however, they did not contribute to the overall flux state.

*In silico testing of compatible solute synthesis*

The synthesis of sucrose in constrained and unconstrained *iGd895* was catalyzed by sucrose phosphorylase (i.e., rxn00577, EC 2.4.1.7). However, this reaction was gapfilled in the model to allow for sucrose production. Although the gene and reaction for sucrose synthase was present (i.e., rxn00579), it was not used for sucrose synthesis and was not feasible as an objective function. This indicated a knowledge gap in the sucrose metabolism pathways, and further research is needed to elucidate compatible solute synthesis in *G. dulcis*.

*In silico* *analysis of knockouts and condition-dependent growth*

We found 10 inessential reactions that each somewhat lowered the growth rate when knocked out in the constrained model, but not the unconstrained model (**File S4**). These included environmental exchange and transfer reactions (i.e., in or out of the cell) for oxygen, ammonia, and xanthine. The other inessential reactions were involved in carbon fixation for photosynthetic organisms (i.e., rxn00102 and rxn00785) or glycolysis/gluconeogenesis pathways (i.e., rxn00459 and rxn001106). These inessential reactions may play a role in supporting growth under specific environmental conditions or in providing alternative pathways for energy production, but were not critical for overall growth in the constrained model. Their identification highlighted the complexity of metabolic networks in cyanobacteria and the potential for redundancy in certain pathways.

*Comparison of metabolic states with global modules*

**Figure S3** illustrates the cumulative variance explained by the principal components (PCs) of the flux spaces in both the unconstrained and constrained simulations. The first 42 PCs accounted for 99.9% of the flux space variation in both simulations (**Fig. S3**, **File S6**), with a relatively even distribution of variance among these PCs. Consequently, the lines representing the variance for both simulations appear indistinguishable in **Figure S3**. The highest variance explained by a PC was 5.65% for the unconstrained simulation and 5.42% for the constrained simulation. On average, each of the first 42 PCs explained 2.38±0.23% of the variation for both flux spaces (± standard error of the mean). The remaining PCs, 269 and 260 for the unconstrained and constrained simulations, respectively, accounted for less than 1% of the variation.

As per Sarathy et al., we condensed the reactions within each PC such that only high-loading reactions explained the variance of each PC ^2^. These high-loading reactions formed a "module," and a group of such modules was referred to as a "global module." From a biochemical standpoint, a global module represented the sets of reactions that substantially contributed to the homeostasis of a specific metabolic state. In our study, basis rotation of the PCs resulted in two global modules, one for each metabolic network (i.e., the unconstrained and constrained simulations).

Each global module for *iGd895* comprised 41 individual modules, which collectively contained a total of 92 and 91 high-loading reactions for the unconstrained and constrained simulations, respectively. All of the modules contained a unique set of reactions, however, several of the reactions took part in more than one module. To illustrate these connections, we used ComMet to construct a reaction map from the reactions within the modules (**Fig. S4**). The constrained network had 826 edges and four unconnected reactions, while the unconstrained network had 848 edges and only one unconnected reaction. Aside from the unconnected reactions, both networks had one highly connected subset with at least 818 reactions, and one small subset composed of four reactions (**Fig. S4**).

We found that the majority of the reactions were involved in a single module, while a small proportion (11-12%) participated in more than one module. In both networks, only around 10 reactions were present in two to a maximum of four modules (**Fig. S4**). These multi-module reactions contributed to multiple PCs in the flux space, suggesting that they were responsible for regulating multiple aspects of network behavior. This complexity presented a challenge for disentangling their effects. However, the vast majority of reactions regulated only a single aspect of network behavior.

Although the number of modules and reactions was similar between the networks, the module structure differed between the simulations. In particular, the unconstrained network had a higher percentage of modules representing a single pathway (61%), while nearly all modules in the constrained network (95%) included multiple pathways (**File S7 and S8**). This indicated that the metabolic network's regulation in response to water limitation involved a change in its modular organization. The greater overlap of pathways within modules in the constrained network suggested a higher degree of interdependence between pathways in response to water limitation.

**References**

1. Dong, M.-J., Luo, H. & Gao, F. Ori-Finder 2022: A Comprehensive Web Server for Prediction and Analysis of Bacterial Replication Origins. *Genomics Proteomics Bioinformatics* (2022) doi:10.1016/j.gpb.2022.10.002.

2. Sarathy, C. *et al.* Comparison of metabolic states using genome-scale metabolic models. *PLOS Comput. Biol.* **17**, e1009522 (2021).
