# Supplementary material for: Unveiling metabolic pathways involved in the extreme desiccation tolerance of an Atacama cyanobacterium
Source: Sci Rep. 2023 Sep 22;13:15767. doi: 10.1038/s41598-023-41879-8 (PMC10516996; doi:10.1038/s41598-023-41879-8)
Supplement: Supplementary file 13 — Supplementary Table S1. [file 41598_2023_41879_MOESM13_ESM.docx]

**Table S1** Initiator recognition sequences (DnaA boxes) in the *G. dulcis* genome predicted by Ori-Finder 2022 via MEME Suite 5.5.0.

| **Motif:**  TGKGRAAAACT | **P-value** | **Site** |
| --- | --- | --- |
|  | 1.66×10^-7^ | TGCAGATTTC-TGGGGAAAACT-TTGGGAAAAT |
|  | 5.10×10^-7^ | TGGGAAAATC-TGTGGAAAACT-CTTCAGTTTA |
|  | 5.91×10^-7^ | CTTCAGTTTA-CGGGGAAAACT-ACTAATGAGT |
|  | 2.02×10^-6^ | AAAAATACTG-TGTGAAAAACT-CTTCAGTTTT |
|  | 2.02×10^-6^ | TTGATTAGAT-TGTGAAAAACT-TGCAGATTTC |
| **Motif:**  GTWTWTSCAMARKTTWTWSWCAGCCWA | 1.26×10^-13^ | AAACTCTTCA-GTTTTTCCACAAGTTTTCCACAGCCAA-TAAGGGTTAA |
|  | 9.58×10^-11^ | GGTTGTTAAA-GTTTTTCCACAGTTTCCACAGCCCCTA-CTACTACTGT |
|  | 9.58×10^-11^ | TTTGTAGTTA-GTATGTGATAAGTTTTTTCCCAGCCAA-CTTTGAGACT |
|  | 3.78×10^-10^ | TTGAGACTTT-GCAACTGCACAGCTTAATGTCAGCTTA-AAAAAATATT |
|  | 1.78×10^-9^ | TCTTAGTAGT-CTTTATGCAACAGATATGGTCAGCAAA-TGGTACGATA |
| **Motif:**  GCTTCTRWGC | 2.83×10^-7^ | GTACGATAGT-GCTTCTGAGC-TAGATAAAAC |
|  | 6.02×10^-7^ | TAAATAATAA-GCTTCTATGC-ATCCTTAAAA |
