# Supplementary material for: Unveiling metabolic pathways involved in the extreme desiccation tolerance of an Atacama cyanobacterium
Source: Sci Rep. 2023 Sep 22;13:15767. doi: 10.1038/s41598-023-41879-8 (PMC10516996; doi:10.1038/s41598-023-41879-8)
Supplement: Supplementary file 14 — Supplementary Table S2. [file 41598_2023_41879_MOESM14_ESM.docx]

| **Name** | **KEGG Map ID** | **Reaction count** | **Metabolite count** |
| --- | --- | --- | --- |
| Metabolic pathways | map01100 | 1895 | 1836 |
| Biosynthesis of secondary metabolites | map01110 | 1032 | 1391 |
| Microbial metabolism in diverse environments | map01120 | 957 | 931 |
| Fatty acid biosynthesis | map00061 | 183 | 50 |
| Purine metabolism | map00230 | 179 | 100 |
| Amino sugar and nucleotide sugar metabolism | map00520 | 141 | 114 |
| Steroid hormone biosynthesis | map00140 | 131 | 100 |
| Arginine and proline metabolism | map00330 | 126 | 93 |
| Glycosphingolipid biosynthesis - lacto and neolacto series | map00601 | 121 | 52 |
| Pyrimidine metabolism | map00240 | 112 | 75 |
| Carotenoid biosynthesis | map00906 | 111 | 114 |
| Porphyrin and chlorophyll metabolism | map00860 | 105 | 126 |
| Tyrosine metabolism | map00350 | 102 | 78 |
| Aminobenzoate degradation | map00627 | 93 | 85 |
| Tryptophan metabolism | map00380 | 92 | 81 |
| Methane metabolism | map00680 | 91 | 84 |
| Starch and sucrose metabolism | map00500 | 90 | 63 |
| Glycerophospholipid metabolism | map00564 | 88 | 58 |
| Phenylpropanoid biosynthesis | map00940 | 87 | 65 |
| Fatty acid degradation | map00071 | 85 | 57 |
| Metabolism of xenobiotics by cytochrome P450 | map00980 | 84 | 121 |
| Cysteine and methionine metabolism | map00270 | 83 | 59 |
| Polycyclic aromatic hydrocarbon degradation | map00624 | 82 | 105 |
| Diterpenoid biosynthesis | map00904 | 82 | 99 |
| Sesquiterpenoid and triterpenoid biosynthesis | map00909 | 81 | 85 |
| Isoquinoline alkaloid biosynthesis | map00950 | 81 | 94 |
| Fructose and mannose metabolism | map00051 | 79 | 55 |
| Phenylalanine metabolism | map00360 | 79 | 74 |
| Chlorocyclohexane and chlorobenzene degradation | map00361 | 77 | 80 |
| Benzoate degradation | map00362 | 74 | 67 |
| Glyoxylate and dicarboxylate metabolism | map00630 | 74 | 60 |
| Flavonoid biosynthesis | map00941 | 73 | 68 |
| Pentose and glucuronate interconversions | map00040 | 72 | 55 |
| N-Glycan biosynthesis | map00510 | 71 | 36 |
| Pyruvate metabolism | map00620 | 71 | 31 |
| Glycine, serine and threonine metabolism | map00260 | 68 | 50 |
| Nicotinate and nicotinamide metabolism | map00760 | 67 | 49 |
| Lysine degradation | map00310 | 64 | 52 |
| Glutathione metabolism | map00480 | 63 | 42 |
| Propanoate metabolism | map00640 | 62 | 44 |
| **Name (Cont.)** | **KEGG Map ID (Cont.)** | **Reaction count (Cont.)** | **Metabolite count**  **(Cont.)** |
| Drug metabolism - cytochrome P450 | map00982 | 62 | 89 |
| Galactose metabolism | map00052 | 60 | 47 |
| Various types of N-glycan biosynthesis | map00513 | 59 | 38 |
| Butanoate metabolism | map00650 | 59 | 41 |
| Phenylalanine, tyrosine and tryptophan biosynthesis | map00400 | 58 | 35 |
| Arachidonic acid metabolism | map00590 | 58 | 75 |
| Anthocyanin biosynthesis | map00942 | 58 | 66 |
| Glucosinolate biosynthesis | map00966 | 58 | 75 |
| Glycolysis / Gluconeogenesis | map00010 | 57 | 31 |
| Ascorbate and aldarate metabolism | map00053 | 57 | 47 |
| Limonene and pinene degradation | map00903 | 57 | 64 |
| Valine, leucine and isoleucine degradation | map00280 | 56 | 47 |
| Nitrogen metabolism | map00910 | 56 | 42 |
| Pentose phosphate pathway | map00030 | 55 | 38 |
| Inositol phosphate metabolism | map00562 | 55 | 44 |
| Ubiquinone and other terpenoid-quinone biosynthesis | map00130 | 54 | 64 |
| Benzoate degradation via CoA ligation | map00632 | 54 | 57 |
| Sulfur metabolism | map00920 | 54 | 44 |
| Fatty acid elongation | map00062 | 53 | 41 |
| Alanine, aspartate and glutamate metabolism | map00250 | 53 | 29 |
| Biosynthesis of type II polyketide products | map01057 | 53 | 136 |
| Carbon fixation pathways in prokaryotes | map00720 | 52 | 51 |
| Primary bile acid biosynthesis | map00120 | 51 | 51 |
| Isoflavonoid biosynthesis | map00943 | 49 | 63 |
| Cyanoamino acid metabolism | map00460 | 48 | 45 |
| Terpenoid backbone biosynthesis | map00900 | 48 | 43 |
| Steroid biosynthesis | map00100 | 47 | 49 |
| Methionine metabolism | map00271 | 47 | 31 |
| Toluene degradation | map00623 | 47 | 43 |
| Naphthalene degradation | map00626 | 47 | 60 |
| Flavone and flavonol biosynthesis | map00944 | 47 | 49 |
| Androgen and estrogen metabolism | map00150 | 46 | 46 |
| Monoterpenoid biosynthesis | map00902 | 45 | 49 |
| Sphingolipid metabolism | map00600 | 44 | 25 |
| beta-Alanine metabolism | map00410 | 42 | 31 |
| Chloroalkane and chloroalkene degradation | map00625 | 41 | 40 |
| Histidine metabolism | map00340 | 40 | 45 |
| Xylene degradation | map00622 | 40 | 38 |
| Glycosaminoglycan degradation | map00531 | 39 | 23 |
| **Name (Cont.)** | **KEGG Map ID (Cont.)** | **Reaction count (Cont.)** | **Metabolite count**  **(Cont.)** |
| Glycosphingolipid biosynthesis - ganglio series | map00604 | 38 | 31 |
| Biosynthesis of unsaturated fatty acids | map01040 | 38 | 80 |
| Glycerolipid metabolism | map00561 | 37 | 30 |
| Lysine biosynthesis | map00300 | 36 | 35 |
| Vitamin B6 metabolism | map00750 | 34 | 32 |
| Folate biosynthesis | map00790 | 34 | 35 |
| Tropane, piperidine and pyridine alkaloid biosynthesis | map00960 | 34 | 68 |
| Aminoacyl-tRNA biosynthesis | map00970 | 34 | 52 |
| Other types of O-glycan biosynthesis | map00514 | 33 | 0 |
| Carbon fixation in photosynthetic organisms | map00710 | 33 | 34 |
| Pantothenate and CoA biosynthesis | map00770 | 33 | 28 |
| Biosynthesis of 12-, 14- and 16-membered macrolides | map00522 | 32 | 84 |
| Biotin metabolism | map00780 | 32 | 28 |
| Drug metabolism - other enzymes | map00983 | 32 | 39 |
| Fluorobenzoate degradation | map00364 | 31 | 32 |
| Mucin type O-Glycan biosynthesis | map00512 | 30 | 15 |
| Ether lipid metabolism | map00565 | 30 | 23 |
| Retinol metabolism | map00830 | 30 | 24 |
| Cutin, suberine and wax biosynthesis | map00073 | 29 | 34 |
| Caffeine metabolism | map00232 | 29 | 21 |
| One carbon pool by folate | map00670 | 29 | 9 |
| Citrate cycle (TCA cycle) | map00020 | 28 | 23 |
| Valine, leucine and isoleucine biosynthesis | map00290 | 27 | 23 |
| Butirosin and neomycin biosynthesis | map00524 | 27 | 33 |
| Lipopolysaccharide biosynthesis | map00540 | 27 | 30 |
| Stilbenoid, diarylheptanoid and gingerol biosynthesis | map00945 | 27 | 25 |
| Linoleic acid metabolism | map00591 | 25 | 30 |
| Thiamine metabolism | map00730 | 25 | 31 |
| alpha-Linolenic acid metabolism | map00592 | 24 | 40 |
| Nitrotoluene degradation | map00633 | 24 | 27 |
| Caprolactam degradation | map00930 | 24 | 22 |
| Selenocompound metabolism | map00450 | 23 | 28 |
| Styrene degradation | map00643 | 23 | 24 |
| Brassinosteroid biosynthesis | map00905 | 23 | 29 |
| Indole diterpene alkaloid biosynthesis | map00403 | 22 | 33 |
| Taurine and hypotaurine metabolism | map00430 | 22 | 22 |
| Phosphonate and phosphinate metabolism | map00440 | 22 | 48 |
| Glycosphingolipid biosynthesis - globo series | map00603 | 22 | 17 |
| **Name (Cont.)** | **KEGG Map ID (Cont.)** | **Reaction count (Cont.)** | **Metabolite count**  **(Cont.)** |
| Dioxin degradation | map00621 | 22 | 58 |
| C5-Branched dibasic acid metabolism | map00660 | 22 | 32 |
| Riboflavin metabolism | map00740 | 22 | 23 |
| Aflatoxin biosynthesis | map00254 | 21 | 27 |
| Peptidoglycan biosynthesis | map00550 | 21 | 35 |
| Atrazine degradation | map00791 | 21 | 21 |
| Bisphenol degradation | map00363 | 19 | 25 |
| Fluorene degradation | map00628 | 19 | 36 |
| Geraniol degradation | map00281 | 18 | 19 |
| Streptomycin biosynthesis | map00521 | 18 | 24 |
| Glycosylphosphatidylinositol(GPI)-anchor biosynthesis | map00563 | 17 | 17 |
| Indole alkaloid biosynthesis | map00901 | 17 | 47 |
| Glycosaminoglycan biosynthesis - chondroitin sulfate / dermatan sulfate | map00532 | 15 | 14 |
| Betalain biosynthesis | map00965 | 15 | 33 |
| Tetracycline biosynthesis | map00253 | 14 | 22 |
| Penicillin and cephalosporin biosynthesis | map00311 | 14 | 18 |
| D-Glutamine and D-glutamate metabolism | map00471 | 14 | 12 |
| Biosynthesis of type II polyketide backbone | map01056 | 14 | 15 |
| Ethylbenzene degradation | map00642 | 13 | 14 |
| Zeatin biosynthesis | map00908 | 13 | 49 |
| Insect hormone biosynthesis | map00981 | 13 | 25 |
| DDT degradation | map00351 | 12 | 20 |
| Novobiocin biosynthesis | map00401 | 12 | 34 |
| Polyketide sugar unit biosynthesis | map00523 | 12 | 37 |
| Glycosaminoglycan biosynthesis - heparan sulfate / heparin | map00534 | 12 | 7 |
| Steroid degradation | map00984 | 12 | 16 |
| D-Arginine and D-ornithine metabolism | map00472 | 11 | 10 |
| Carbazole degradation | map00629 | 11 | 18 |
| Biosynthesis of ansamycins | map01051 | 9 | 32 |
| Benzoxazinoid biosynthesis | map00402 | 8 | 9 |
| Puromycin biosynthesis | map00231 | 7 | 12 |
| Synthesis and degradation of ketone bodies | map00072 | 6 | 6 |
| Clavulanic acid biosynthesis | map00331 | 6 | 10 |
| D-Alanine metabolism | map00473 | 6 | 6 |
| Lipoic acid metabolism | map00785 | 6 | 6 |
| Furfural degradation | map00365 | 5 | 12 |
| Biosynthesis of vancomycin group antibiotics | map01055 | 5 | 19 |
| **Name (Cont.)** | **KEGG Map ID (Cont.)** | **Reaction count (Cont.)** | **Metabolite count**  **(Cont.)** |
| Biosynthesis of siderophore group nonribosomal peptides | map01053 | 4 | 13 |
| Acridone alkaloid biosynthesis | map01058 | 3 | 7 |
